# Matrix Metalloproteinase Inhibitors (MMPIs) from Marine Natural Products: the Current Situation and Future Prospects

**DOI:** 10.3390/md7020071

**Published:** 2009-03-31

**Authors:** Chen Zhang, Se-Kwon Kim

**Affiliations:** 1 Department of Chemistry, Pukyong National University, Busan, 608-737, Republic of Korea; E-mail: ahxczc@yahoo.cn; sknkim@pknu.ac.kr; 2 Marine Bioprocess Research Center, Pukyong National University, Busan, 608-737, Republic of Korea; E-mail: sknkim@pknu.ac.kr

**Keywords:** Matrix metalloproteinases (MMPs), Matrix metalloproteinase inhibitors (MMPIs), Tissue inhibitors of metalloproteinase (TIMPs), Marine natural products, NF-κB, AP-1

## Abstract

Matrix metalloproteinases (MMPs) are a family of more than twenty five secreted and membrane-bound zinc-endopeptidases which can degrade extracellular matrix (ECM) components. They also play important roles in a variety of biological and pathological processes. Matrix metalloproteinase inhibitors (MMPIs) have been identified as potential therapeutic candidates for metastasis, arthritis, chronic inflammation and wrinkle formation. Up to present, more than 20,000 new compounds have been isolated from marine organisms, where considerable numbers of these naturally occurring derivatives are developed as potential candidates for pharmaceutical application. Eventhough the quantity of marine derived MMPIs is less when compare with the MMPIs derived from terrestrial materials, huge potential for bioactivity of these marine derived MMPIs has lead to large number of researches. Saccharoids, flavonoids and polyphones, fatty acids are the most important groups of MMPIs derived from marine natural products. In this review we focus on the progress of MMPIs from marine natural products.

## 1. Introduction

Matrix metalloproteinases (MMPs) are a family of zinc-dependent endopeptidases that degrade extracellular matrix (ECM) components and play important roles in a variety of biological and pathological processes [1]. MMPs regulate the synthesis and secretion of cytokines, growth factors, hormone receptors, and cell adhesion molecules. They also contribute to the growth and development, morphogenesis, tissue remodeling, angiogenesis, arthritis, cardiovascular disease, stroke, multiple sclerosis, neurodegenerative diseases, allergies, as well as cancer and a series of physiological and pathological processes [2, 3]. In tumor progression MMPs are important not only in invasion, angiogenesis, and metastasis, but also MMPs have roles in cancer cells transformation, growth, apoptosis, signal transduction and immune regulation [4, 5]. Therefore, the development of matrix metalloproteinase inhibitors (MMPIs) to treat some important diseases, including cancers, neurodegenerative diseases, cardiovascular diseases and various kinds of inflammatory diseases have broad prospects [3–9].

Generally MMPs consist of a propeptide domain having about 80 amino acids, a catalytic metalloproteinase domain of about 170 amino acids, a linker peptide of variable lengths (also called the “hinge region”) and a hemopexin domain of about 200 amino acids [10]. However, not all of these domains are essential for MMPs; some MMPs lack the linker peptide and the hemopexin domain. MMPs contain a Zn^2+^ catalytic core; this zinc-binding site has a conservative HEXXHXXGXXH amino acid sequence. The catalytic domains of MMPs show homology, as their three-dimensional (3D) structure of the enzyme active site are highly conservative. The catalytic domain includes a pocket called the“S1′ pocket” located to the right of the zinc atom. This pocket is hydrophobic in nature, but variable in depth depending on the MMP. It is therefore one of the determining factors of substrate specificity of MMPs. Accordingly, the S1′ pocket in the catalytic domains of MMPs is most notable, and its depth, as well as the length and amino acid sequence of the peptide which around the S1′ pocket is important basis for design and synthesis of the MMPIs [11–13].

MMPs’ activities can be regulated by endogenous inhibitors, such as tissue inhibitors of metalloproteinase (TIMPs), α_2_-macroglobin, heparin and the reversion-inducing cysteine-rich protein with kazal motifs (RECK) [4, 5]. There are four TIMPs in humans (TIMP-1, -2, -3 and -4) with 22–29 kDa. TIMP-1 and TIMP-3 are glycoproteins, but TIMP-2 and TIMP-4 do not contain carbohydrates. They inhibit all MMPs tested so far [14]. These four TIMPs have different expression and distribution in the tissue and may be responsible for regulating the activity of a large number of protease families *in vivo*, including the metalloproteinases of a disintegrin and metalloproteinases (ADAMs) family. However, the TIMPs and other endogenous inhibitors have diversity of biological functions and also the protein delivery techniques are not developed, the use of these endogenous inhibitors in clinical applications have been delayed [4, 5].

Design and synthesis of MMPIs has gone through several stages of development over the past 20 years [13]. Originally MMPIs was designed by simulating MMPs substrate, this is the first-generation of MMPIs. Most of them are peptides and their derivatives. Inhibition is occurred by chelating the Zn^2+^ of the MMP by the group present in inhibitors, such as hydroxylamine, carboxyl, SH, etc.. Mainly the Zn^2+^ is chelated with the oxyammonia-base by combining the substrate analogs peptide, at the same time through the substrate analogs peptide combine with the catalytic domains of MMP, and thus plays the inhibitory effect. Strong Zn^2+^ chelating agents such as hydroxamate as a class of MMPIs have been developed, representative of these inhibitors are the British Biotech’s Batimastat (BB-94) and Marimastat (BB-2516), and they all have ideal inhibitory activity with the MMPs. Even through, these compounds can interact with Zn^2+^; they can’t distinguish between different MMPs. Therefore, the uses of first-generation of MMPIs as drugs in clinical applications were restricted. Their shortcomings include: poor selectivity of MMPs, hydroxylamine substances have low oral bioavailability, the metabolism is not stable, poor solubility and the drug toxicity increase after amelioration. Therefore it was strongly suggested that the first generation of MMPIs must use another group in place of hydroxylamine group as a Zn^2+^ chelating group, or design new non-peptide MMPIs. For these proposed MMPIs, first, lead compounds were selected through high-throughput screening, then these lead compounds are reformed with the Safety Analysis Report (SAR) guidance, finally these new reagents with better effect was formed. The second-generation MMPIs also contain Zn^2+^ chelating group. These drugs have eliminated some of shortcomings of peptide drugs with considerable selectivity towards MMPs. However, in clinical applications they also have been impeded due to effectiveness and side effects [15, 16].

Clinical trials for the anti-cancer and anti-arthritis effects have been carried out using many early MMPIs. However, only a few MMPIs were effective (such as Marimastat, the overall survival rate of the gastric cancer and pancreatic cancer patients increase). Therefore they have not been used in the later stages of clinical trials. At present, only one MMPI (Periostat) is being used clinically for periodontitis therapy [5, 15].

With intensive studies on MMPs, the MMPs host-cell defense functions and physiological functions have been discovered by researchers. The early MMPIs whether peptide inhibitors or small molecule inhibitors, their activities are most dependent on the Zn^2+^ chelating group and MMPs S1′ pocket combined group. However the Zn^2+^ chelating group also reduces these early MMPIs’ selectivity. In addition, these early MMPIs inhibit some MMPs physiological functions and some other metalloprotease such as DPP III and leucine aminopeptidase, when they inhibit the abnormal MMPs in pathology situation [5, 17].

To sum up the above arguments, the clinical trials of MMPIs in broad-spectrum, face the obstacle, as well as the normal physiological functions of MMPs should be further studied for the choice of drugs which are selectively acting on them for the MMPs relevant diseases. MMPs S1′ pocket determine the specificity of substrates and inhibitors in a large extent, therefore the S1′ pocket is very important for the design and synthesis of MMPIs. Design of MMPIs should be based on the unique functions of MMPs S1′ pocket, not only to increase the selectivity for this MMP, but also greatly reduce the inhibition of other class of metalloprotease such as ADAMs. At present, development of the new generation of MMPIs is guided by this idea. In addition, development of new type of MMPIs with different inhibiting mechanisms can increase the drugs’ selectivity; which may play a key role in the treatment of various diseases related to MMPs [18–21].

Broadly speaking, the mechanisms of inhibiting the activity of MMPs include, direct inhibition of the enzymes, blocking the MMPs proenzyme activation, suppressing the synthesis of MMPs in the gene level, and so on. The MMPIs can be divided into four classes: the natural MMPIs secreted by tissues; synthetic MMPIs; MMPIs screened from natural products and the MMPIs screened from the phage display random peptide library and antibody library. The synthetic MMPIs and natural product derived MMPIs are the hot spots. In recent years, due to the synthetic small molecule inhibitors meet a variety of issues in clinical applications, more attention is given to the research of MMPIs derived from natural products.

Lots of successful research work have been conducted to identify MMPIs from land natural products, also got a lot of results. For instance, Kim *et al.* were screened for nearly 90 kinds of extracts from clinical application herbal medicines, and found that the extracts from Baicalin, Cinnamon, Euonymus, and Magnolia have strong inhibitory effects on MMPs [22–24]. However we should not forget that the ocean is treasure house which is full of natural products with amazing biological and pharmacological activities. About 80% of the planet’s animal and plant growth in the ocean, and the variety of marine bacteria can reach 500–100 million. Therefore discovering the ideal MMPIs from marine natural products is a very hot topic at present. The leitmotiv along this review is to sum up the progress of research work carried out on identifying MMPIs from marine natural products. We divided the marine derived MMPIs into three classes, marine saccharoid MMPIs, marine flavonoids and polyphenols MMPIs and marine fatty acid MMPIs, and their properties will be discussed in this review.

## 2. MMPIs from marine natural products

### 2.1. Marine saccharoid MMPIs

The marine saccharoid MMPIs are very popular among marine derived MMPIs area. The most of marine saccharoid MMPIs inhibit MMP by direct down-regulation of MMP-9 transcription or via inhibition of activator protein-1(AP-1) pathway or nuclear factor κB (NF-κB) pathway. Kim *et al*. report the inhibitory effect of chitooligosaccharides (COS) on activation and expression of matrix metalloproteinase-2 (MMP-2) in primary human dermal fibroblasts (HDFs) for the first time. COS with 3–5 kDa exhibited the highest inhibitory effect on MMP-2 activity in HDFs, and protein expression of MMP-2 was also inhibited by COS with same molecular weight. This inhibition was caused by the decrease in gene expression and transcriptional activity of MMP-2[25]. Quang *et al.* have investigated the effect of Chitooligosaccharides (COS) on activity and expression of MMP-9 in HT1080 cells by gelatin zymography, RT-PCR, gene reporter assay, and western blot analysis. They found that MMP-9 inhibition in the presence of COS was clearly observed in gelatin zymography. Specifically, 1- to 3-kDa COS (COS-I) exhibited the highest inhibitory effect on MMP-9 activity in HT1080 cells among tested molecular mass fractions. It was also found that COS-I was capable of inhibiting both gene and protein expression of MMP-9 (P<0.01) [26]. The novel low molecular-weight carboxylated Chitooligosaccharides (CCOS) has been evaluated for MMP-9 inhibitory effect on human fibrosarcoma cell line [27]. A clear dose-dependent inhibition on MMP-9 mediated gelatinolytic activities were observed in HT1080 cells following the treatment with CCOS in zymography experiments. Transfection studies carried out with MMP-9 and AP-1 reporter constructs suggested that the observed reduction in MMP-9 expression was due to down-regulation of MMP-9 transcription which mediated via inhibition of AP-1. However, in the presence of CCOS, NF-κB and TIMP-1 expression levels remained constant [27].

Adriana *et al.* investigated on the shrimp heparin-like glycosaminoglycan isolated from *L. vannamei* which was able to interfere on MMP-9 activity in activated human leukocytes. And it has the capacity to reduce 90% MMP-9 activity, either in a lower or higher concentrations (10 and 100 μg/mL), with pronounced effects [28]. In present studies, sulfated glucosamine (SG) has been reported to relieve joint pain and inflammation in many arthritis patients. Niranjan *et al.* studied for SG inhibitory effects on MMP-2 and MMP-9 in human fibrosarcoma cells. Expression and activity of above MMPs studied suggested SG as a potent MMP inhibitor, and inhibition of MMP-2 and MMP-9 was due to down-regulation of transcription factor, NF-κB. However, expression of activator protein-1 (AP-1) was not affected by SG treatment. Moreover, down-regulation of NF-κB resulted in production of low levels of both NF-κB p50 and p65 proteins and directly affected activation process of MMP-2 and MMP-9 expressions [29].

Angiogenesis is involved in initiating and promoting several diseases such as cancer and cardiovascular events. Chen et al. obtained highly sulfated λ-carrageenan oligosaccharides (λ-CO) by carrageenan depolymerization. They have demonstrated that λ-carrageenan oligosaccharides could effectively inhibit angiogenesis in the CAM (chick chorioallantoic membrane) model and human umbilical vein endothelial cells (HUVECs). Significant inhibition of vessel growth was observed at 200 μg/pellet. A histochemistry assay also revealed a decrease of capillary plexus and connective tissue in λ-CO treated samples. λ-CO inhibited the viability of cells at the high concentration of 1 mg/mL, whereas it affected the cell survival slightly (>95%) at a low concentration (<250 μg/mL). Furthermore, the inhibitory action of λ-CO was also observed in the endothelial cell invasion and migration at relatively low concentrations (150 300 μg/mL), through down-regulation of intracellular matrix metalloproteinases (MMP-2) expression on endothelial cells. [30].

Wang *et al.* isolated the sulfated S. maindroni ink polysaccharide (SIP-SII) from cuttlefish *Sepiella maindroni*, and examined the effects of SIP-SII on the expression of matrix metalloproteinases MMP-2 and MMP-9 as well as tumor cell invasion and migration. SIP-SII (0.8–500 mg/ml) significantly decreased the expression of MMP-2 activity in human ovarian carcinoma cells SKOV3. No significant decrease of MMP-9 was detected in the cell line after SIP-SII treatment [31].

Fucoidan is a uniquely-structured sulfated polysaccharide found in the cell walls of several types of brown seaweed which has been recently evaluated for its bioactivities by Ye *et al*. [32]. Enzyme-digested fucoidan extracts prepared from seaweed, Mozuku of Cladosiphon novae-caledoniae kylin showed in vitro invasion and angiogenesis abilities of human tumor cells. The mechanism of significant inhibition of HT1080 cells invasion by fucoidan extracts, possibly via suppressing MMP-2 and MMP-9 activities. Further, they investigated the effects of the fucoidan extracts on angiogenesis of human uterine carcinoma HeLa cells, and found that fucoidan extracts suppressed expression and secretion of vascular endothelial growth factor (VEGF) [32].

Marine saccharoid MMPIs exhibit high MMPs inhibitory activity either by direct inhibition of the enzyme or by inhibiting the expression of MMPs. And also these marine saccharoid MMPIs have shown low toxicity levels. However, due to high molecular weight of theses MMPIs the structure-activity relationship and also the mechanism of the activities are hard to be addressed by the researchers. If these shortcomings are overcome in the future, marine saccharoid MMPIs have a great potential to be used in clinical applications.

### 2.2. Marine flavonoids and polyphenols MMPIs

Flavonoid glycosides, isorhamnetin 3-O-b-D-glucosides, and quercetin 3-O-b-D-glucoside were isolated from *Salicornia herbacea* and their inhibitory effects on matrix metalloproteinase-9 and -2 were evaluated in human fibrosarcoma cell line [31]. These flavonoid glycosides led to the reduction of the expression levels and activities of MMP-9 and -2 without any significant difference between these flavonoid glycosides in zymography experiments. Protein expression levels of both MMP-9 and MMP-2 were inhibited and TIMP-1 protein level was enhanced by these flavonoid glycosides [33].

Kim *et al*., for the first time, report a detailed study on the inhibitory effects of phlorotannins in brown algae, *Ecklonia cava* (EC) on MMP activities. A novel gelatin digestion assay could visualize complete inhibition of bacterial collagenase-1 activity at 20 μg/ml of EC extract during preliminary screening studies. Sensitive fluorometric assay revealed that EC extract can specifically inhibit both MMP-2 and MMP-9 activities significantly (P<0.001) at 10 μg/ml. In addition, artificially induced activities of MMP-2 and MMP-9 in human dermal fibroblasts and HT1080 cells were inhibited by EC extract in a more or less similar manner to the positive control doxycycline. More interestingly, EC extract did not exert any cytotoxic effect even at 100 μg/ml, anticipating, its potential use as a safe MMP inhibitor [34].

The active compound from methanol extracts prepared from roots of *Rhodiola sacra* has been identified as 3-(3, 4-dihydroxy-phenyl)-acrylic acid phenethyl ester (caffeic acid phenethyl ester, CAPE) [35, 36]. And Lee *et al*. found that these active compounds can down-regulate enhanced MMP -9 activities [37].

Joe *et al*. examined the inhibitory effects of 29 seaweed extracts on transcriptional activities of MMP-1 expression. And found that the eckol and dieckol from *Ecklonia species* have showed strong inhibition of both NF-κB and AP-1 reporter activity, which were well correlated with their abilities to inhibit MMP-1 expression. In addition, MMP-1 expression was dramatically attenuated by treatment with the eckol or dieckol [38].

Matrix metalloproteinases (MMPs), a key component in photoaging of the skin due to exposure to ultraviolet A, appear to be increased by UV-irradiation-associated generation of reactive oxygen species (ROS). Ryu et al. demonstrates that the alga *Corallina pilulifera* methanol extract which has been shown a high phenolic content, reduced the expression of UV-induced MMP-2 and -9 in human dermal fibroblast by dose dependently manner, and has also antioxidant activity capable of strongly inhibiting free radicals [39].

In murine asthma model, Kim et al. observed that MMP-9 expression was significant reduced via the administration of *Ecklonia cava* extracts. And *Ecklonia cava* extracts reveal Suppressor of cytokine signaling-3 (SOCS-3) expression and a reduction in the increased eosinophil peroxidase (EPO) activities. Their results indicate that *Ecklonia cava* extracts may prove to be a useful therapeutic agent for the treatment of ovalbumin -induced asthma [40].

The compounds eckol, 2dieckol, 6,6′-bieckol and 1-(3′,5′-dihydroxyphenoxy) -7-(2″,4″,6″-trihydroxy-phenoxy)-2,4,9-trihydroxydibenzo-1,4,-dioxin were extracted from brown algae, *Ecklonia cava*, and Ryu et al. have investigated these compounds inhibited the proinflammatory cytokines induced expression of MMP-1, -3 and -13 [41].

Flavonoids and polyphenols MMPIs have excellent MMPs inhibitory activities; however they show a high toxicity level. Therefore, the pharmaceutical applications of these MMPIs are limited. Researchers should pay attention to reduce their toxicity levels by altering the structure in a way by preserves it’s bioactivity. Then this class of MMPIs will gain a huge potential to be used in clinical applications

### 2.3. Marine fatty acid MMPIs

Researchers have identified that the long-chain fatty acids could inhibit MMPs. however for different MMPs the degree of inhibition is different, such as oleic acid, elaidic acid can inhibit MMP-2 and MMP-9 with the micromol Ki values, although their inhibitory effects on collagenase-1 (MMP-1) are weak, as assessed using synthetic or natural substrates [42]. The fatty acid chain length and its degree of saturation is related to the level of inhibition, as the fatty acids with long carbon chains showed stronger inhibition than the short ones, and the nonsaturation degree showed a positive correlation to the overall inhibitory capacity of the fatty acid chains [42,43]. Fatty acids also bind to neutrophil elastase, the parinaric acids, fluorescent-conjugated tetraenoic fatty acids of plant origin, are inhibitors of neutrophil elastase. cis-Parinaric acid (cis-PA) interacts with the enzyme in two inhibitory modes. The high affinity interaction (Ki = 55 +/− 6 nM) results in partial noncompetitive inhibition of amidolytic activity, with 82% residual activity. A lower affinity interaction with cis-PA (Ki = 4 +/− 1 microM) results in competitive inhibition [44, 45]. the fatty acids also bind to plasmin, such as The ability of oleic acid to modulate fibrinolysis was measured by following the urokinase-mediated and plasminogen-dependent cleavage of 125I-labelled fibrin clots. Oleic acid levels within the physiological range exerted a concentration-dependent inhibition of urokinase-mediated fibrinolytic activity [46, 47], and some other serine proteinases, meanwhile modulate their catalytic activities.

It is well known that the marine fishes are rich in omega-3 long-chain polyunsaturated fatty acids (ω3 LC-PUFAs), especially eicosapentaenoic acid (EPA) and docosahexaenoic acid (DHA), which are active nutrients [48]. Suzuki *et al*. found that the inhibition of lung metastasis of a colon cancer cell line by EPA and DHA was associated with a reduced activity of MMP-9, however MMP-2 activity was not affected by the diet containing PUFAs [49, 50]. The MMP-9 activity was reduced by in uterus, placenta and liver tissues of rat fed diets enriched with DHA, with a decreased activity of MMP-2 [50]. They explained their finding by a competition of the ω3 LC-PUFAs with arachidonic acid for incorporation into membrane phospholipids. This would consequently change the production of prostaglandin PGE2 and thereby affect on MMP activities.

Acetylenic fatty acids isolated from marine sponges have exhibited wide range of biological activities such as cytotoxicity [51], antimicrobial [52] and antifouling [53] activities, and enzyme inhibition [54]. Callysponginol sulfate A is the first sulfated C_24_ acetylenic fatty acid from marine organisms. Fujita *et al*. 2002 extracted the sodium 1-(12-hydroxy)octadecanyl sulfate from an ascidian collected in western Japan, inhibited MMP-2 in 2002. And both natural and synthetic forms inhibited MMP-2 with an IC_50_ value at 9.0 μg/mL; thus the stereochemistry of the hydroxyl group did not influence the activity [55]. And after one year, they reported another compound, callysponginol sulfate A, a new sulfated C_24_ acetylenic fatty acid, extracted from the marine sponge, *Callyspongia truncate* [56]. This compound inhibited recombinant MT1-MMP with an IC_50_ value at 15.0 μg/mL, however the desulfated callysponginol sulfate A did not show any inhibitory activity against MT1-MMP. Considering this result as well as the similar activity of structurally unrelated sulfated compounds, the MT1-MMP inhibition activity is probably a consequence of the sulfate [56].

### 2.4. Other marine natural products MMPIs

Shark cartilage extracts researches are very popular in recently [57]. The compounds extracted from shark cartilage (such as NeovastatÒ, AE-941, U-995 etc.) have been investigated on their potential use as MMPIs. These compounds were analyzed with regard to their anti-angiogenic and antimetastatic effects on the activity of several MMPs [58], because MMPs are intimately connected with angiogenetic and metastatic processes. The results revealed that NeovastatÒ inhibits enzymatic activity of MMP-2 with minor inhibition of MMP-1, -7, -9 and -13. And also interestingly the western blot analysis evidenced the presence of TIMP-like proteins within AE-941, could be responsible for its specific MMP inhibitory property [59]. The tissue inhibitors of metalloprotease 1, 2 and 3 (TIMP-1, -2, -3) and tumor suppressor protein genes have been cloned and characterized from shark cartilage extracts [52, 60, 61].

Alkaloid Ageladine A extract from the marine sponge, *Agelas nakamurai*, and Ageladine A inhibited not only MMP-2, but also MMPs-1, -8, -9, -12, and -13 with IC_50_ values of 2.0, 1.2, 0.39, 0.79, 0.33, and 0.47 μg/mL, respectively, while its N-methylated derivatives did not inhibit MMP-2. As we know that many potent MMP inhibitors are known to bind with Zn^2+^ in the catalytic domain. But Ageladine A was not capable to chelate Zn^2+^. Moreover, the kinetic analysis indicated that inhibition of MMP-2 by Ageladine A was not competitive when judged in the Lineweaver-Burk plot. Thus, the inhibition mechanism of Ageladine A was presumed to be unique [62].

The Atlantic cod (Gadus morhua) muscle contains a 21-kDa proteinase inhibitor. The inhibitor had properties similar to human TIMP-2. The inhibitor was found to inhibit the gelatin-degrading enzymes present in the gelatin-bound fraction. In addition, it inhibited gelatinolytic activity obtained from a human macrophage cell medium rich in MMP-9 [63].

(+)-Aeroplysinin-1, an antibacterial brominated compound produced by certain sponges, was selected during a blind high-throughput screening as new potential antiangiogenic compounds obtained from marine organisms. The concentration of MMP-2 in the medium conditioned by aeroplysinin-treated cells was clearly lower than that in untreated cell medium. The MMP-2 bands in aeroplysinin-treated cell conditioned media were 60 + 4% compared to those of untreated cells, whereas extracts of treated cells yielded MMP-2 bands that were almost twofold (1.77 + 0.04) those of untreated cells. Thus, aeroplysinin-1 seems to affect mainly the release of MMP-2 to the medium [64].

## 3. Conclusions

The marine environment is characterized by high biodiversity offering vast variety of natural products which could be used as potential drugs, particularly in the area of cancer chemotherapy, such like the matrix metalloproteinase inhibitors. Therefore continuation of finding new leads in this area of extracting bioactivity compounds from marine natural products will make much sense.

MMPIs design and synthesis has been done for ages and has gone through several development stages. Although many of the synthetic inhibitors of MMPs showed good inhibitory activity, however, the compounds do not have an ideal MMPs selectivity, combined with others limitations such as the low oral bioavailability, unstable metabolism, biological toxicity, and also these inhibitors in clinical trials show excessive side effects. Due to these major shortcomings this type of MMPIs failed to be used as drugs [3, 5, 65].

With MMPIs finding of functionality of MMP’s in normal physiology functions, the development of MMPIs entered a new period [66, 67]. In recent years, the non-metal chelating agent class of MMPIs reports has begun to appear. Isolating MMPs from marine natural products has been gradually gained more attention. Some marine natural products have been isolated with MMPs inhibitory activities and further, some compounds have special restraint or high selectivity [68]. Such as Ageladine A which inhibit MMP-2 was not competitive judging from the Lineweaver-Burk plot. Thus, the inhibition mechanism of Ageladine A was presumed to be unique [62]. These MMPIs will be the focus of future work.

## Abbreviations

MMPs: matrix metalloproteinases
ECM: extracellular matrix
MMPIs: matrix metalloproteinase inhibitors
TIMPs: tissue inhibitors of metalloproteinase
RECK: reversion-inducing cysteine-rich protein with kazal motifs
ADAMs: a disintegrin and metalloproteinases
SAR: safety analysis report
COS: chitooligosaccharides
HDFs: human dermal fibroblasts
CCOS: carboxylated chitooligosaccharides
SGlc: sulfated glucosamine
NF-κB: nuclear factor κB
AP-1: activator protein-1
λ-CO: λ-carrageenan oligosaccharides
HUVECs: human umbilical vein endothelial cells
SIP-SII: sulfated S. maindroni ink polysaccharide
EC: Ecklonia cava
